# Adaptive Neural Network Robust Control of FOG with Output Constraints

**DOI:** 10.3390/biomimetics10060372

**Published:** 2025-06-05

**Authors:** Shangbo Liu, Baowang Lian, Jiajun Ma, Xiaokun Ding, Haiyan Li

**Affiliations:** 1 School of Electronics and Information, Northwestern Polytechnical University, 127 West Youyi Road, Beilin District, Xi’an 710072, China; bwlian@nwpu.edu.cn; 2 Electronic and College of Big Data and Communication Engineering Information Engineering, Guizhou University, Guiyang 550025, China; jjma3@gzu.edu.cn; 3 AVIC Flight Automatic Control Research Institute, Northwestern Polytechnical University, 127 West Youyi Road, Beilin District, Xi’an 710072, China; dingxiaokun@aliyun.com; 4 School of Life Science and Technology, Changchun University of Science and Technology, Changchun 130022, China; lihaiyan@cust.edu.cn

**Keywords:** fiber optic gyroscope, neural network, adaptive control, output constraints

## Abstract

In this work, an adaptive robust control method based on Radial Basis Function Neural Network (RBFNN) is proposed. Inspired by the local response characteristics of biological neurons, this method can reduce the influence of nonlinear errors and unknown perturbations in the extreme working conditions of the aircraft, such as high dynamics and strong vibration, so as to achieve high tracking accuracy. In this method, the dynamic model of the nonlinear error of the fiber optic gyroscope is proposed, and then the unknown external interference observer is designed for the system to realize the estimation of the unknown disturbances. The controller design method combines the design of the adaptive law outside the finite approximation domain of the achievable condition design of the sliding mode surface, and adjusts the controller parameters online according to the conditions satisfied by the real-time error state, breaking through the limitation of the finite approximation domain of the traditional neural network. In the finite approximation domain, an online adaptive controller is constructed by using the universal approximation ability of RBFNN, so as to enhance the robustness to nonlinear errors and external disturbances. By designing the output constraint mechanism, the dynamic stability of the system is further guaranteed under the constraints, and finally its effectiveness is verified by simulation analysis, which provides a new solution for high-precision inertial navigation.

## 1. Introduction

The optical fiber gyroscope (FOG) is an interferometric angular rate sensor based on the Sagnac effect, constructed from a ring of optical fibers. When the optical fiber ring rotates around its normal, two coherent light beams traveling in opposite directions within the ring will produce a phase difference proportional to the angular rotational rate. By detecting changes in the interference intensity caused by this phase difference, the angular rate can be measured. The FOG is the second generation of optical gyroscope after laser gyroscope, which has become the core device of high-precision navigation system for various aircraft because of its advantages of fast start-up, high precision, high bandwidth, all-solid-state, and strong environmental adaptability, and has been widely used in sea, land, air, space and other fields [1,2,3,4,5], and its performance directly affects the navigation accuracy of aircraft. High-precision FOGs typically adopt a fully digital closed-loop processing scheme, using Y-waveguide integrated optical phase modulators as core components (multifunction integrated optic circuit, MIOC), and introducing a feedback control loop to compensate in real time for the phase difference caused by rotation around zero using the Y-waveguide, thereby forming a closed-loop control system. This scheme can significantly improve the dynamic range and nonlinearity of the scale factor of the fiber optic gyroscope. The closed-loop fiber optic gyroscope relies on the performance of the control system to stably track the input angular velocity, and the performance of the control system directly affects the dynamic output accuracy of the FOG. However, with the rapid development of aviation and aerospace technology, the emergence of new aircraft such as super-aircraft and the continuous improvement of the flight ability of the aircraft, the FOG will work in a long-term extreme dynamic environment accompanied by high dynamics, strong vibration and impact, and the fiber optic gyroscope will be subject to nonlinear errors and disturbance interference, and the tracking performance of its closed-loop control system will be greatly limited, resulting in the aggravation of the dynamic output error of the FOG, which directly affects the inertial navigation error and causes catastrophic consequences.

In order to improve the closed-loop control performance of FOGs in harsh flight environments, all parties have made this effort. In [6], a high-precision real-time detection and closed-loop control method of FOG loop gain was proposed, and the closed-loop automatic control loop of the gain was used to achieve the stable maintenance of the loop gain at the preset reference optimal value. In order to eliminate the steady-state error and appropriately increase the system bandwidth in [7], the proposed digital controller adds a proportional link to the original integration link to improve the dynamic characteristics of the FOG. In [8], in order to eliminate the steady-state error, an integral link and a moving average filter are added to the digital controller, and a proportional integral derivative (PID) control algorithm is introduced to improve the system dynamics. The research in the above literature shows that the traditional PID controller has achieved certain improvements in eliminating steady-state errors, suppressing deviation changes, and improving response speed. However, in the process of FOG, the vibration or other interference factors of the instrument will cause the characteristic parameters or results of the FOG to change, and the control strategy of using the fixed parameters of the traditional PID controller often leads to poor control effect. Therefore, in order to cope with the special environmental changes, many scholars have adopted fuzzy logic control to further optimize and improve the parameters of the PID controller to improve the stability and robustness of the controller, so as to better meet the control needs of the gyroscope in the process of operation and ensure the stability and reliability of the control effect, as described in. A novel F-PID composite controller combined with fuzzy control was proposed in [8]. The deviation value of input and output and the change rate of deviation are input language variables, and the parameters of PID are output language variables, so as to realize online adjustment of PID parameters and improve the static and dynamic characteristics of FOGs. However, the number of fuzzy rules involved in the design of the controller is large, and the design of the fuzzy rules and membership function is entirely based on experience, so the design of the controller is relatively difficult. In [9], the improved BP neural network was used to adjust and optimize the parameters of the common PID, and the influence of the previous output value of the PID on the output value of the PID was suppressed through active series correction, which solved the problems of local extreme value and slow convergence speed of the BP neural network. In [10], the improved gray wolf algorithm is used to tune the traditional PID parameters, and the Euclidean distance rate of change is used to dynamically adjust the convergence factor to balance the global search ability of the algorithm. The dynamic adaptive weight factor is introduced to improve the optimization speed and accuracy of the algorithm. Although the controller in Refs. [8,9,10] can achieve the desired control effect, the optimization process is complex, computationally intensive, and time-consuming. In [11], a fuzzy PID controller based on the gravitational search algorithm is used to realize the closed-loop control of FOG, which has advantages in dynamic performance optimization, but the algorithm is optimized globally through the gravitational search algorithm, but its parameter adjustment requires multiple trial and error, and after coupling with the parameters of fuzzy PID, the overall optimization process takes a long time, which limits its practicability. In [12,13], a hybrid control system is designed by combining the advantages of fuzzy logic system and neural network, but due to its high interpretability and learning ability, the computational complexity is high, which leads to an increase in the amount of online computation, and it is difficult to use it in a system with high real-time performance.

In recent years, with the maturity of artificial neural network technology, Radial Basis Function Neural Network (RBFNN) is a kind of feedforward neural network based on local approximation theory, which has attracted great attention from researchers in various fields due to its simple structure, strong nonlinear mapping ability and fast learning speed. In [14], RBFNN was used to compensate for the bias error of the laser gyroscope, and the approximation effect of the RBFNN algorithm on the nonlinear error model was verified, and the improved RBFNN algorithm had the best performance. In [15,16,17,18,19], the RBFNN dynamic identification of the dynamic characteristics of MEMS gyroscopes and the approximation of external disturbance are used to ensure that the control system can reach the sliding surface from any initial state and converge to the equilibrium point in a limited time by introducing a sliding mode controller, reducing the number of parameters and avoiding the problem of parameter expansion. Finally, the simulation results show that in the presence of model uncertainty and external interference, RBFNN optimization control can reduce the input flutter and improve the timeliness and effectiveness of tracking.

The design inspiration comes from the local receptive fields and global information integration mechanisms in biological neural systems, which are inherently consistent with the multi-channel parallel processing characteristics of insect compound eyes in biologically inspired visual systems [20,21,22]. This simulates the ability of biological neurons to locally approximate nonlinear disturbances, achieving autonomous parameter optimization of the FOG in high-dynamic environments and carries out the simulation verification of the related control algorithms, so that the FOG control system can quickly realize high-precision closed-loop control under nonlinear error and uncertain disturbances.

## 2. Control System Model of FOG

**Figure 1:**
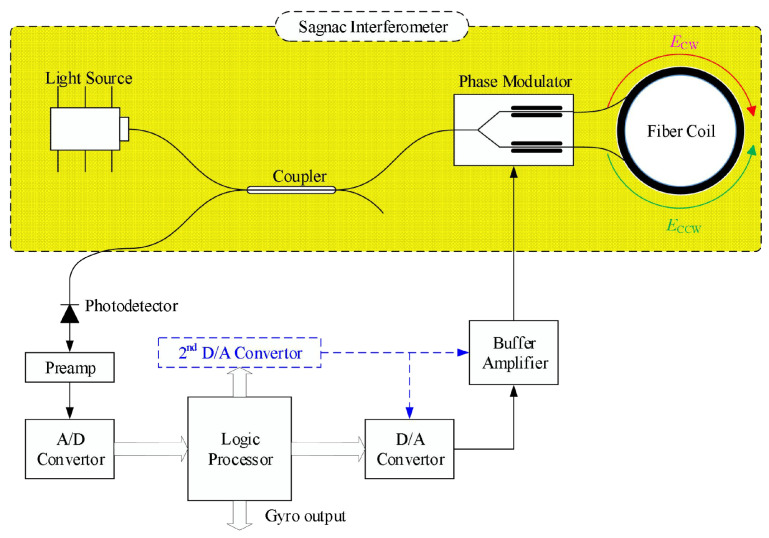
Structure diagram of the FOG System.

The structure of the digital closed-loop FOG is shown in Figure 1. The optical section includes the light source, coupler, phase modulator, optical fiber loop, and photodetector, while the electrical section comprises a preamplifier, analog-to-digital converter (ADC), logic processor, digital-to-analog converter (DAC), and its output buffer amplifier, among others. The light source, coupler, phase modulator, and optical fiber loop form the Sagnac interferometer. When the fiber optic loop rotates, a non-reciprocal phase shift is generated between the counterpropagating light waves in the interferometer. The phase shift $\Phi_{s}$ is proportional to the angular velocity Ω, with the scale factor given by $K_{s}$ = $\approx 2pi\frac{LD}{\lambda c}$, where L is the length of the optical fiber loop, D is the average diameter of the fiber loop, λ is the average wavelength of the light source, and c is the speed of light in a vacuum.

In the digital closed-loop FOG, The light emitted from the light source is split into two beams traveling in opposite directions after passing through the coupler and *Y* waveguide. The *Y* waveguide integrates beam-splitting, polarization, and phase modulation functions, and is therefore also known as a multifunction integrated optic circuit (MIOC). These two beams propagate in clockwise and counterclockwise directions within the fiber ring and interfere at the beam-splitting port of the Y waveguide. The spatial rotation angular velocity induces a phase difference between the two beams, which leads to changes in the interference light intensity. This interference signal is then output through the coupler to the photodetector. By processing the detected light intensity signal, the corresponding angular velocity can be extracted. To ensure stable operation of the fiber optic gyroscope at the optimal response point and to improve the linearity of the scale factor and the anti-interference capability, a closed-loop feedback mechanism is implemented. Leveraging the phase modulation functionality of the MIOC, a compensating phase is applied in real time to cancel out the phase shift caused by rotation, thereby stabilizing the operating point at a zero differential phase state and enabling precise computation of the angular velocity based on the feedback signal. This achieves high linearity and low noise in angular rate measurement. Moreover, to maintain accuracy, the feedback loop must operate within its linear range. If the phase difference exceeds 2π, nonlinear errors may occur due to the system’s inability to accurately track the actual phase change. To address this, periodic 2π-resetting is employed to keep the feedback signal within the linear region, thus enhancing both the accuracy and stability of the measurement.

The modulation phase generated by the *Y*-waveguide compensates for the phase induced by the Sagnac effect. Considering interference factors such as the nonlinear modulation effects of the *Y*-waveguide, the control model of the digital closed-loop FOG is given as follows:

(1) $$\left\{\begin{array}{c} {\ddot{\phi }}_{F}+k_{1}\cos\left(\phi_{s}-\phi_{F}\right){\dot{\phi }}_{F}+k_{2}\sin\left(\phi_{s}-\phi_{F}\right)+D\left(t\right)+u=0\\ y=\phi_{F} \end{array}\right.$$

where $\phi_{F}={\left[\phi_{F1},\phi_{F2},\phi_{F3}\right]}^{T}$ represents the system state, $\phi_{F1}$, $\phi_{F2}$, $\phi_{F3}$ denote the location on XYZ axis, respectively, $\phi_{s}={\left[\phi_{s1},\phi_{s2},\phi_{s3}\right]}^{T}$ denotes XYZ axis, respectively, $k_{1}=\mathrm{diag}\left\{k_{11},k_{12},k_{13}\right\}$, $k_{2}=\mathrm{diag}\left\{k_{21},k_{22},k_{23}\right\}$, $k_{ij}$ the known signal vector of the system (with i = 1, 2 and j = 1, 2, 3), $D\left(t\right)={\left[D_{1}\left(t\right),D_{2}\left(t\right),D_{3}\left(t\right)\right]}^{T}$ represents the unknown external disturbance, and *u* is the controller to be designed. $y=\phi_{F}={\left[y_{1},y_{2},y_{3}\right]}^{T}$ represents the system output. The system (1) satisfies the following conditions:

**Assumption A1.**  *The external disturbance $D_{j}\left(t\right)$, is assumed to be unknown but bounded, satisfying the condition, $|D_{i}\left(t\right)|\leq {\bar{D}}_{i}$, where ${\bar{y}}_{dj}$ is a known positive constant.*

**Assumption A2.**  *The desired trajectory $y_{d}={\left[y_{d1},y_{d2},y_{d3}\right]}^{T}$ is bounded and needs to be tracked by the location of the FOG system (1). This implies that the desired trajectory satisfies the condition $|y_{dj}|\;\leq \;{\bar{y}}_{dj}$, where ${\bar{y}}_{dj}$ is a known positive constant.*

**Remark 1.**  *The disturbances and trajectories considered in this paper are indeed bounded. While unbounded disturbances pose a significant challenge due to the severe shocks they may introduce, which could potentially render the control ineffective, it is important to note that even in such cases, the functions remain bounded within a defined finite domain. This ensures that the system behavior can still be analyzed and controlled within practical limits.*

The above analysis indicates that under 2π-reset conditions, the modulation state lacks symmetry, resulting in strong coupling between the primary and secondary closed-loop controls in the digital closed-loop FOG. The symmetry of the modulation state is the fundamental condition for decoupling the primary closed-loop control and the secondary closed-loop control in the digital closed-loop FOG.

### Design of the RBFNN Controller

**Figure 2:**
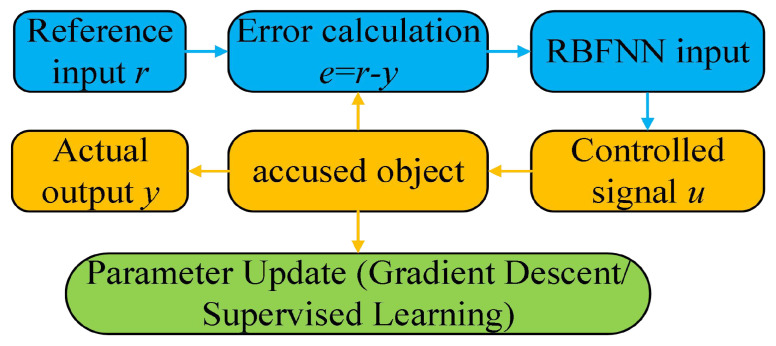
Structural schematic diagram of the RBFNN controller.

The basic structure schematic diagram of the RBFNN controller is shown in Figure 2.

(2) $$\left\{\begin{array}{c} {\dot{x}}_{1}=x_{2}={\dot{\phi }}_{F}\\ {\dot{x}}_{2}={\ddot{x}}_{1}={\ddot{\phi }}_{F}=-k_{1}\cos\left(\phi_{s}-x_{1}\right)x_{2}-k_{2}\sin\left(\phi_{s}-x_{1}\right)-u-D\left(t\right) \end{array}\right.$$

where the state vector is $x_{1}={\left[x_{11},x_{12},x_{13}\right]}^{T}$, $x_{2}={\left[x_{21},x_{22},x_{23}\right]}^{T}$, $h_{a1}\leq x_{11}\leq h_{a2}$, $h_{c1}\leq x_{13}\leq h_{c2}$, $h_{a1}$, $h_{a2}$, $h_{b1}$, $h_{b2}$, $h_{c1}$, and $h_{c2}$ denote the bounded values. *u* is the controller signal in Figure 2 that will be designed.

In Figure 2, the core parts of the RBFNN controller are the input layer, the controlled layer and the output layer; The controlled object module receives the controlled object u and outputs t parameters of the actual response signal y; In the parameter adjustment module, it adjusts the weights and basis function RBFNN based on error back propagation.

In this paper, let the signal $y_{d}={\left[y_{d1},y_{d2},y_{d3}\right]}^{T}$ represent the desired signal, that is tracked by the position of state vector $x_{1}$$\mu_{1}=x_{1}-y_{d}$, $\mu_{2}=x_{2}-\beta_{1}={\left[\mu_{21},\mu_{22},\mu_{23}\right]}^{T}$, where $\beta_{1}$ is the virtual controller to be designed. Then, it has ${\dot{\mu }}_{1}={\dot{x}}_{1}-{\dot{y}}_{d}$, Take the Lyapunov function as:

(3) $$V_{1}=\frac{1}{2}\log\frac{h_{a1}^{2}}{h_{a1}^{2}-\mu_{11}^{2}}+\frac{1}{2}\log\frac{h_{a2}^{2}}{h_{a2}^{2}-\mu_{12}^{2}}+\frac{1}{2}\log\frac{h_{a3}^{2}}{h_{a3}^{2}-\mu_{13}^{2}}$$

The derivative of function (3) is:

(4) $$\begin{array}{c} {\dot{V}}_{1}=\frac{\mu_{11}{\dot{\mu }}_{11}}{h_{a}^{2}-\mu_{11}^{2}}+\frac{\mu_{12}{\dot{\mu }}_{12}}{h_{b}^{2}-\mu_{12}^{2}}+\frac{\mu_{13}{\dot{\mu }}_{13}}{h_{c}^{2}-\mu_{13}^{2}}\;\\ =\left[\frac{\mu_{11}}{h_{a}^{2}-\mu_{11}^{2}},\frac{\mu_{12}}{h_{b}^{2}-\mu_{12}^{2}},\frac{\mu_{13}}{h_{c}^{2}-\mu_{13}^{2}}\right]\left(\mu_{2}+\beta_{1}-{\dot{y}}_{d}\right) \end{array}$$

Thus, the virtual controller can be designed as $\beta_{1}={\dot{y}}_{d}-r\mu_{1}$, where $r=\mathrm{diag}\{r_{1},r_{2},r_{3}\}$$r_{1}$ denote positive real constant, so it obtains:

(5) $$\begin{array}{c} {\dot{V}}_{1}=-\frac{r_{1}\mu_{11}^{2}}{h_{a}^{2}-\mu_{11}^{2}}-\frac{r_{2}\mu_{12}^{2}}{h_{b}^{2}-\mu_{12}^{2}}-\frac{r_{3}\mu_{13}^{2}}{h_{c}^{2}-\mu_{13}^{2}}\;\\ +\frac{\mu_{11}\mu_{21}}{h_{a}^{2}-\mu_{11}^{2}}+\frac{\mu_{12}\mu_{22}}{h_{b}^{2}-\mu_{12}^{2}}+\frac{\mu_{13}\mu_{23}}{h_{c}^{2}-\mu_{13}^{2}} \end{array}$$

Because ${\dot{\mu }}_{2}={\dot{x}}_{2}-{\dot{\beta }}_{1}=-k_{1}\cos\left(\phi_{s}-x_{1}\right)x_{2}-k_{2}\sin\left(\phi_{s}-x_{1}\right)-u-D\left(t\right)-{\dot{\beta }}_{1}$, selecting the following Lyapunov function:

(6) $$V_{2}=V_{1}+\frac{1}{2}\mu_{2}^{T}\mu_{2}$$

(7) $${\dot{V}}_{2}={\dot{V}}_{1}+\mu_{2}^{T}\left[-k_{1}\cos\left(\phi_{s}-x_{1}\right)x_{2}-k_{2}\sin\left(\phi_{s}-x_{1}\right)-u-D\left(t\right)-{\dot{\beta }}_{1}\right]$$

Design the interference observer:

(8) $$\hat{D}=P\left(t\right)+h\left(\mu_{2}\right)\mu_{2}$$

(9) $$\dot{P}\left(t\right)=h\left(\mu_{2}\right)\left[k_{1}\cos\left(\phi_{s}-x_{1}\right)x_{2}+k_{2}\sin\left(\phi_{s}-x_{1}\right)+u+\hat{D}+{\dot{\beta }}_{1}\right]$$

where $P\left(t\right)={\left[P_{1}\left(t\right),P_{2}\left(t\right),P_{3}\left(t\right)\right]}^{T},\;h\left(\mu_{2}\right)=\mathrm{diag}\left\{h_{1}\left(\mu_{2}\right),h_{2}\left(\mu_{2}\right),h_{3}\left(\mu_{2}\right)\right\}$. Because $\tilde{D}=\hat{D}-D$, then it has:

(10) $$\begin{array}{c} \dot{\tilde{D}}=\dot{D}-\dot{\hat{D}}=\dot{P}\left(t\right)+h\left(\mu_{2}\right){\dot{\mu }}_{2}-\dot{D}\\ =h\left(\mu_{2}\right)\left[k_{1}\cos\left(\phi_{s}-x_{1}\right)x_{2}+k_{2}\sin\left(\phi_{s}-x_{1}\right)+u+\hat{D}+{\dot{\beta }}_{1}\right]\\ +h\left(\mu_{2}\right)\left[-k_{1}\cos\left(\phi_{s}-x_{1}\right)x_{2}-k_{2}\sin\left(\phi_{s}-x_{1}\right)-u-D\left(t\right)-{\dot{\beta }}_{1}\right]-\dot{D}\\ =h\left(\mu_{2}\right)\tilde{D}-\dot{D} \end{array}$$

From (10), it knows

(11) $${\dot{\mu }}_{2}=-k_{1}\cos\left(\phi_{s}-x_{1}\right)x_{2}-k_{2}\sin\left(\phi_{s}-x_{1}\right)-u-\hat{D}-{\dot{\beta }}_{1}$$

Let the nonlinear function be $-k_{1}\cos\left(\phi_{s}-x_{1}\right)x_{2}-k_{2}\sin\left(\phi_{s}-x_{1}\right)-{\dot{\beta }}_{1}=\Delta \;$ At this point, the observers in Equations (8) and (9) contain unknown nonlinear terms, which cannot be directly applied. Therefore, it is necessary to redesign the observer based on the Radial Basis Function Neural Network (RBFNN). This paper considers the design of observers and controllers under two scenarios: when the states $\mu_{2}$ are outside the RBFNN universal approximation domain and when they are within the approximation domain.

**Theorem 1.**  *Under the conditions of Assumptions 1 and 2, for system *(1)*, with the controller $u=O_{3\times 1}$, disturbance observer *(12)*, and adaptive laws *(13)* and (*14*), the extended state vector $z={\left[\mu_{2}^{T},\rho ,\vartheta^{T},\varepsilon^{T},W_{j}^{T}\right]}^{T}$ will reach the sliding surface $s_{1}$, $s_{2}$*

(12) $$\hat{D}=P\left(t\right)+h\left(\mu_{2}\right)\mu_{2},\;\dot{P}\left(t\right)=h\left(\mu_{2}\right)\left[\bar{\Delta }+u+\hat{D}\right]$$

*The following form of adaptive law is designed:*

(13) $$\dot{\rho }=\left\{\begin{array}{cc} \frac{1}{\alpha^{2}\rho }\left(L+\Xi \right), & ∥z∥\geq ∥\mu_{2}∥>\alpha |\rho |\\ -\frac{1}{\alpha^{2}\rho }\left(L+\Xi \right), & ∥z∥\geq \alpha |\rho |>∥\mu_{2}∥ \end{array}\right.$$

(14) $${\dot{\hat{\vartheta }}}_{j}=0,\;{\dot{\hat{\varepsilon }}}_{j}=0,\;{\dot{\hat{W}}}_{j}=O_{1\times n}$$

*where $\Xi =\frac{|\mu_{11}\left|\cdot \right|\mu_{21}|}{|h_{a}^{2}-\mu_{11}^{2}|}+\frac{|\mu_{12}\left|\cdot \right|\mu_{22}|}{|h_{b}^{2}-\mu_{12}^{2}|}+\frac{|\mu_{13}\left|\cdot \right|\mu_{23}|}{|h_{c}^{2}-\mu_{13}^{2}|}+∥\mu_{2}∥(\bar{\Delta }+|\hat{D}\left|\right)$.*

**Proof.**  Case 1: From the state vector $z={\left[\mu_{2}^{T},\rho ,\vartheta^{T},\varepsilon^{T},W_{j}^{T}\right]}^{T}$, it is evident $∥z∥\;\geq \;∥\mu_{2}∥$ that when $∥z∥\;\geq \;∥\mu_{2}∥\;>\alpha |\rho |$ satisfies the given condition, the sliding surface can be chosen in the following form:

(15) $$s_{1}=V_{1}+\frac{1}{2}\mu_{2}^{T}\mu_{2}-\frac{1}{2}\alpha^{2}\rho^{2}+\sum_{j=1}^{3}\frac{1}{2\gamma_{1j}}{\tilde{\vartheta }}_{j}^{2}+\sum_{j=1}^{3}\frac{1}{2\delta_{1j}}{\tilde{\varepsilon }}_{j}^{2}+\sum_{j=1}^{3}\frac{1}{2\chi_{1j}}{\tilde{W}}_{j}^{T}{\tilde{W}}_{j}+\sum_{j=1}^{3}\frac{1}{2\tau_{1j}}{\tilde{D}}_{j}^{2}$$

Order $\tilde{V}=\frac{1}{2}s_{1}^{2}$, there is:

(16) $$\begin{array}{c} \dot{\tilde{V}}=s_{1}{\dot{s}}_{1}=s_{1}\{{\dot{V}}_{1}+\mu_{2}^{T}\left[\Delta -u-\hat{D}\right]-\alpha^{2}\rho \dot{\rho }+\gamma_{1j}^{-1}\sum_{j=1}^{3}{\tilde{\vartheta }}_{j}{\dot{\hat{\vartheta }}}_{j}\\ +\delta_{1j}^{-1}\sum_{j=1}^{3}{\tilde{\varepsilon }}_{j}{\dot{\hat{\varepsilon }}}_{j}+\chi_{1j}^{-1}\sum_{j=1}^{3}{\tilde{W}}_{j}^{T}{\dot{\hat{W}}}_{j}+\tau_{1j}^{-1}\sum_{j=1}^{3}{\tilde{D}}_{j}{\dot{\hat{D}}}_{j} \end{array}$$

Using open-loop control $u=O_{3\times 1}$, the following conclusions are valid:

(17) $$\begin{array}{c} \dot{\tilde{V}}\leq s_{1}\left\{-\frac{r_{1}\mu_{11}^{2}}{h_{a}^{2}-\mu_{11}^{2}}-\frac{r_{2}\mu_{12}^{2}}{h_{b}^{2}-\mu_{12}^{2}}-\frac{r_{3}\mu_{13}^{2}}{h_{c}^{2}-\mu_{13}^{2}}+\frac{\mu_{11}\mu_{21}}{h_{a}^{2}-\mu_{11}^{2}}+\frac{\mu_{12}\mu_{222}}{h_{b}^{2}-\mu_{12}^{2}}+\frac{\mu_{13}\mu_{23}}{h_{c}^{2}-\mu_{13}^{2}}+\mu_{2}^{T}\left[\Delta -\hat{D}\right]\right\}\;\\ -\alpha^{2}\rho \dot{\rho }+\gamma_{1j}^{-1}\sum_{j=1}^{3}{\tilde{\vartheta }}_{j}{\dot{\hat{\vartheta }}}_{j}+\delta_{1j}^{-1}\sum_{j=1}^{3}{\tilde{\varepsilon }}_{j}{\dot{\hat{\varepsilon }}}_{j}+\chi_{1j}^{-1}\sum_{j=1}^{3}{\tilde{W}}_{j}^{T}{\dot{\hat{W}}}_{j}+\tau_{1j}^{-1}\sum_{j=1}^{3}{\tilde{D}}_{j}{\dot{\hat{D}}}_{j}\}\;\\ \leq s_{1}\{\frac{|\mu_{11}\left|\cdot \right|\mu_{21}|}{|h_{a}^{2}-\mu_{11}^{2}|}+\frac{|\mu_{12}\left|\cdot \right|\mu_{22}|}{|h_{b}^{2}-\mu_{12}^{2}|}+\frac{|\mu_{13}\left|\cdot \right|\mu_{23}|}{|h_{c}^{2}-\mu_{13}^{2}|}+∥\mu_{2}∥(\bar{\Delta }+|\hat{D}\left|\right)\;\\ -\alpha^{2}\rho \dot{\rho }+\gamma_{1j}^{-1}\sum_{j=1}^{3}{\tilde{\vartheta }}_{j}{\dot{\hat{\vartheta }}}_{j}+\delta_{1j}^{-1}\sum_{j=1}^{3}{\tilde{\varepsilon }}_{j}{\dot{\hat{\varepsilon }}}_{j}+\chi_{1j}^{-1}\sum_{j=1}^{3}{\tilde{W}}_{j}^{T}{\dot{\hat{W}}}_{j}+\tau_{1j}^{-1}\sum_{j=1}^{3}{\tilde{D}}_{j}{\dot{\hat{D}}}_{j}\}\;\\ \leq -s_{1}L \end{array}$$

From conclusion (17), it can be inferred that the extended state *z* will reach the sliding surface $s_{1}$ within finite time.

Case 2: When $∥z∥\;\geq \;\alpha |\rho |\;\geq \;∥\mu_{2}∥$, satisfies the given condition, the sliding surface is designed as:

(18) $$s_{2}=V_{1}+\frac{1}{2}\alpha^{2}\rho^{2}-\frac{1}{2}\mu_{2}^{⊤}\mu_{2}+\sum_{j=1}^{3}\left(\frac{1}{2\gamma_{1j}}{\tilde{\vartheta }}_{j}^{2}+\frac{1}{2\delta_{1j}}{\tilde{\varepsilon }}_{j}^{2}+\frac{1}{2\chi_{1j}}{\tilde{W}}_{j}^{⊤}{\tilde{W}}_{j}+\frac{1}{2\tau_{1j}}{\tilde{D}}_{j}^{2}\right)$$

Order $\tilde{V}=\frac{1}{2}s_{1}^{2}$, there is:

(19) $$\begin{array}{c} \dot{\tilde{V}}=s_{1}{\dot{s}}_{1}=s_{1}\left\{{\dot{V}}_{1}-\mu_{2}^{⊤}\left[\Delta -u-\hat{D}\right]\right\}\\ +\alpha^{2}\rho \dot{\rho }+\gamma_{1j}^{-1}\sum_{j=1}^{3}{\tilde{\vartheta }}_{j}{\dot{\hat{\vartheta }}}_{j}+\delta_{1j}^{-1}\sum_{j=1}^{3}{\tilde{\varepsilon }}_{j}{\dot{\hat{\varepsilon }}}_{j}+\chi_{1j}^{-1}\sum_{j=1}^{3}{\tilde{W}}_{j}^{T}{\dot{\hat{W}}}_{j}+\tau_{1j}^{-1}\sum_{j=1}^{3}{\tilde{D}}_{j}{\dot{\tilde{D}}}_{j} \end{array}$$

Under open-loop control $u=O_{3\times 1}$, the following conclusion holds:

(20) $$\begin{array}{c} \dot{\tilde{V}}\leq s_{2}\left\{-\frac{r_{1}\mu_{11}^{2}}{h_{a}^{2}-\mu_{11}^{2}}-\frac{r_{2}\mu_{12}^{2}}{h_{b}^{2}-\mu_{12}^{2}}-\frac{r_{3}\mu_{13}^{2}}{h_{c}^{2}-\mu_{13}^{2}}+\frac{\mu_{11}\mu_{21}}{h_{a}^{2}-\mu_{11}^{2}}+\frac{\mu_{12}\mu_{22}}{h_{b}^{2}-\mu_{12}^{2}}+\frac{\mu_{13}\mu_{23}}{h_{c}^{2}-\mu_{13}^{2}}-\mu_{2}^{⊤}\left(\Delta -\hat{D}\right)\right\}\;\\ \;+\alpha^{2}\rho \dot{\rho }+\gamma_{1j}^{-1}\sum_{j=1}^{3}{\tilde{\vartheta }}_{j}{\dot{\hat{\vartheta }}}_{j}+\delta_{1j}^{-1}\sum_{j=1}^{3}{\tilde{\varepsilon }}_{j}{\dot{\hat{\varepsilon }}}_{j}+\chi_{1j}^{-1}\sum_{j=1}^{3}{\tilde{W}}_{j}^{T}{\dot{\hat{W}}}_{j}+\tau_{1j}^{-1}\sum_{j=1}^{3}{\tilde{D}}_{j}{\dot{\tilde{D}}}_{j}\\ \;\leq s_{2}\{\frac{|\mu_{11}\left|\cdot \right|\mu_{21}|}{|h_{a}^{2}-\mu_{11}^{2}|}+\frac{|\mu_{12}\left|\cdot \right|\mu_{22}|}{|h_{b}^{2}-\mu_{12}^{2}|}+\frac{|\mu_{13}\left|\cdot \right|\mu_{23}|}{|h_{c}^{2}-\mu_{13}^{2}|}+∥\mu_{2}∥(∥\Delta ∥+∥\hat{D}∥)\}\\ \;+\alpha^{2}\rho \dot{\rho }+\gamma_{1j}^{-1}\sum_{j=1}^{3}{\tilde{\vartheta }}_{j}{\dot{\hat{\vartheta }}}_{j}+\delta_{1j}^{-1}\sum_{j=1}^{3}{\tilde{\varepsilon }}_{j}{\dot{\hat{\varepsilon }}}_{j}+\chi_{1j}^{-1}\sum_{j=1}^{3}{\tilde{W}}_{j}^{T}{\dot{\hat{W}}}_{j}+\tau_{1j}^{-1}\sum_{j=1}^{3}{\tilde{D}}_{j}{\dot{\tilde{D}}}_{j}\}\\ \;\leq -s_{2}L \end{array}$$

Similarly, conclusion (20) ensures that the state *z* will reach the sliding surface $s_{2}$ within finite time. When the extended state vector *z* lies within the finite approximation domain of the RBFNN, i.e., when the conditions $∥\mu_{2}∥\;\leq \;∥z∥\;\leq \;\alpha |\rho |$ are satisfied, the disturbance observer, controller, and adaptive laws are designed in the following form. The disturbance observer is designed to:

(21) $$\hat{D}=P\left(t\right)+h\left(\mu_{2}\right)\mu_{2},\dot{P}\left(t\right)=h\left(\mu_{2}\right)\left[u-\hat{D}-\Phi \left(\frac{X}{\rho }\right)\right]$$

Controller is designed as

(22) $$u={\left[\frac{\mu_{11}\mu_{21}}{h_{a}^{2}-\mu_{11}^{2}},\frac{\mu_{12}\mu_{22}}{h_{b}^{2}-\mu_{12}^{2}},\frac{\mu_{13}\mu_{23}}{h_{c}^{2}-\mu_{13}^{2}}\right]}^{T}+\Phi \left(\frac{X}{\rho }\right)+\bar{k}\mu_{2}-\hat{D}$$

where $\bar{k}=\mathrm{diag}\left\{{\bar{k}}_{1},{\bar{k}}_{2},{\bar{k}}_{3}\right\}$, ${\bar{k}}_{j}$ is the positive real number. And the adaptive law is designed as follows:

(23) $$\dot{\rho }=-\zeta \rho -\sigma \alpha^{2}\sum_{j=1}^{3}({\hat{\vartheta }}_{j}|\rho -1|+\alpha {\hat{\varepsilon }}_{j})\mathrm{sign}\left(\rho \right)$$

(24) $${\dot{\hat{\vartheta }}}_{j}=-\tau_{j}{\hat{\vartheta }}_{j}+\gamma_{2j}\alpha^{2}\left|\rho |\cdot |\rho -1\right|$$

(25) $${\dot{\varepsilon }}_{j}=-\lambda_{j}{\hat{\varepsilon }}_{j}+\delta_{2j}\alpha \left|\rho \right|$$

(26) $${\dot{\hat{W}}}_{j}=-\chi_{2j}\left[\Phi \left(\frac{X}{\rho }\right)\mu_{2}^{T}+\eta_{j}{\hat{W}}_{j}\right]$$

where $\mathrm{sign}\left(\rho \right)=\left\{\begin{array}{cc} 1, & \rho >0\\ -1, & \rho \leq 0 \end{array}\right.$, parameters $\varsigma ,\sigma ,\tau_{j},\lambda_{j},\eta_{j},\gamma_{2j},\delta_{2j},\chi_{2j}$ are some positive constants that are given by the user. Based on (21)–(26), the following Theorem 2 is obtained. □

**Theorem 2.**  *Under the conditions of Assumptions 1 and 2, for system *(1)*, with the disturbance observer *(21)*, controller *(22)*, and adaptive laws *(23)*–*(26)*, the output of system *(1)* can track the desired signal, and all signals in the closed-loop system are uniformly ultimately bounded. Proof: When $∥\mu_{2}∥\;\leq \;∥z∥\;\leq \;\alpha |\rho |$ is true, take*

(27) $$V_{3}=V_{2}+\frac{1}{2\sigma }\rho^{2}+\sum_{j=1}^{3}\frac{1}{2\gamma_{2j}}{\tilde{\vartheta }}_{j}^{2}+\sum_{j=1}^{3}\frac{1}{2\delta_{2j}}{\tilde{\varepsilon }}_{j}^{2}+\sum_{j=1}^{3}\frac{1}{2\chi_{2j}}{\tilde{W}}_{j}^{T}{\tilde{W}}_{j}+\sum_{j=1}^{3}\frac{1}{2\tau_{2j}}{\tilde{D}}_{j}^{2}$$

The differential result is obtained as:

(28) $$\begin{array}{c} {\dot{V}}_{3}={\dot{V}}_{2}+\sigma^{-1}\rho \dot{\rho }+\sum_{j=1}^{3}\frac{1}{\gamma_{2i}}{\tilde{\vartheta }}_{j}{\dot{\hat{\vartheta }}}_{j}+\sum_{j=1}^{3}\frac{1}{\delta_{2i}}{\tilde{\varepsilon }}_{j}{\dot{\hat{\varepsilon }}}_{j}+\sum_{j=1}^{3}\frac{1}{\chi_{2i}}{\tilde{W}}_{j}^{T}{\dot{\hat{W}}}_{j}+\sum_{j=1}^{3}\frac{1}{\tau_{i}}{\tilde{D}}_{j}{\dot{\tilde{D}}}_{j}\\ \;=-\frac{r_{1}\mu_{11}^{2}}{h_{a}^{2}-\mu_{11}^{2}}-\frac{r_{2}\mu_{12}^{2}}{h_{b}^{2}-\mu_{12}^{2}}-\frac{r_{3}\mu_{13}^{2}}{h_{c}^{2}-\mu_{13}^{2}}+\frac{\mu_{11}\mu_{21}}{h_{a}^{2}-\mu_{11}^{2}}+\frac{\mu_{12}\mu_{22}}{h_{b}^{2}-\mu_{12}^{2}}+\frac{\mu_{13}\mu_{23}}{h_{c}^{2}-\mu_{13}^{2}}-\mu_{2}^{T}\left(\Delta -\hat{D}\right)\;\\ +\mu_{2}^{T}\left[\Delta -u-\hat{D}\right]+\sum_{j=1}^{3}\gamma_{2j}^{-1}{\tilde{\vartheta }}_{j}{\dot{\hat{\vartheta }}}_{j}+\sum_{j=1}^{3}\delta_{2j}^{-1}{\tilde{\varepsilon }}_{j}{\dot{\hat{\varepsilon }}}_{j}+\sum_{j=1}^{3}\chi_{2j}^{-1}{\tilde{W}}_{j}^{T}{\dot{\hat{W}}}_{j}+\sum_{j=1}^{3}\tau_{2j}^{-1}{\tilde{D}}_{j}{\dot{\tilde{D}}}_{j} \end{array}$$

According to (22), so it has

(29) $$\begin{array}{c} \mu_{2}^{T}\left[\Delta -\Phi \left(\frac{X}{\rho }\right)\right]\leq ∥\mu_{2}∥(\sum_{j=1}^{3}\vartheta_{j}\alpha |\rho -1|+\varepsilon_{j})\;\\ \leq \alpha |\rho |(\sum_{j=1}^{3}\vartheta_{j}\alpha |\rho -1|+\varepsilon_{j})=\alpha^{2}\sum_{j=1}^{3}(\vartheta_{j}\left|\rho |\cdot |\rho -1|+\alpha |\rho \right|\varepsilon_{j}) \end{array}$$

By (29), using (28) becomes the following form:

(30) $${\dot{V}}_{3}\leq -\frac{r_{1}\mu_{11}^{2}}{h_{a}^{2}-\mu_{11}^{2}}-\frac{r_{2}\mu_{12}^{2}}{h_{b}^{2}-\mu_{12}^{2}}-\frac{r_{3}\mu_{13}^{2}}{h_{c}^{2}-\mu_{13}^{2}}-\bar{k}\mu_{2}^{T}\mu_{2}+\alpha^{2}\sum_{j=1}^{3}\vartheta_{j}\left|\rho |\cdot |\rho -1|+\alpha |\rho \right|\sum_{j=1}^{3}\varepsilon_{j}\;\;+\sigma^{-1}\rho \dot{\rho }+\sum_{j=1}^{3}\gamma_{2j}^{-1}{\tilde{\vartheta }}_{j}{\dot{\hat{\vartheta }}}_{j}+\sum_{j=1}^{3}\delta_{2j}^{-1}{\tilde{\varepsilon }}_{j}{\dot{\hat{\varepsilon }}}_{j}+\sum_{j=1}^{3}\chi_{2j}^{-1}{\tilde{W}}_{j}^{T}{\dot{\hat{W}}}_{j}+\sum_{j=1}^{3}\tau_{2j}^{-1}{\tilde{D}}_{j}{\dot{\tilde{D}}}_{j}$$

Then, the following results can be obtained by the adaptive laws (23)–(26):

(31) $$\begin{array}{c} {\dot{V}}_{3}\leq -\frac{r_{1}\mu_{11}^{2}}{h_{a}^{2}-\mu_{11}^{2}}-\frac{r_{2}\mu_{12}^{2}}{h_{b}^{2}-\mu_{12}^{2}}-\frac{r_{3}\mu_{13}^{2}}{h_{c}^{2}-\mu_{13}^{2}}-\bar{k}\mu_{2}^{T}\mu_{2}+\alpha^{2}\sum_{j=1}^{3}{\hat{\vartheta }}_{j}|\rho |\cdot |\rho -1|+\alpha \sum_{j=1}^{3}\left|\rho \right|{\hat{\varepsilon }}_{j}+\sigma^{-1}\rho \dot{\rho }\;\\ +\sum_{j=1}^{3}\gamma_{2j}^{-1}{\tilde{\vartheta }}_{j}({\dot{\hat{\vartheta }}}_{j}-\gamma_{2j}\alpha^{2}|\rho |\cdot |\rho -1|)+\sum_{j=1}^{3}\delta_{2j}^{-1}{\tilde{\varepsilon }}_{j}({\hat{\varepsilon }}_{j}-\alpha \delta_{2j}|\rho |)\;\\ +\sum_{j=1}^{3}\chi_{2j}^{-1}{\tilde{W}}_{j}\left[-\chi_{2j}\Phi \left(\frac{X}{\rho }\right)\mu_{2}^{T}+\eta {\dot{\hat{W}}}_{j}\right]+\sum_{j=1}^{3}\tau_{2j}^{-1}{\tilde{D}}_{j}{\tilde{D}}_{j}\;\\ \leq -\frac{r\mu_{1}^{2}}{h_{a}^{2}-\mu_{1}^{2}}-\bar{k}\mu_{2}^{T}\mu_{2}-\frac{\zeta }{\sigma }\rho^{2}-\sum_{j=1}^{3}\frac{\tau_{j}}{\gamma_{2j}}{\tilde{\vartheta }}_{j}{\hat{\vartheta }}_{j}-\sum_{j=1}^{3}\frac{\lambda_{j}}{\delta_{2j}}{\tilde{\varepsilon }}_{j}{\hat{\varepsilon }}_{j}-\sum_{j=1}^{3}\frac{\eta_{j}}{\chi_{2j}}{\tilde{W}}_{j}^{T}{\dot{\hat{W}}}_{j}\;\\ +\sum_{j=1}^{3}\frac{1}{2\tau_{2j}}{\tilde{D}}_{j}^{2}+\sum_{j=1}^{3}\frac{1}{2\tau_{2j}}{\bar{\hat{D}}}_{j}^{2} \end{array}$$

In Formula (31), $\mu_{1}^{2}=\mathrm{diag}\left\{\mu_{11}^{2},\mu_{12}^{2},\mu_{13}^{2}\right\}$. Because the following inequalities hold:

(32) $$-\frac{\tau_{j}}{\gamma_{2j}}{\tilde{\vartheta }}_{j}{\hat{\vartheta }}_{j}=-\frac{\tau_{j}}{\gamma_{2j}}{\tilde{\vartheta }}_{j}^{2}-\frac{\tau_{j}}{\gamma_{2j}}{\tilde{\vartheta }}_{j}\vartheta_{j}\leq -\frac{\tau_{j}}{2\gamma_{2j}}{\tilde{\vartheta }}_{j}^{2}+\frac{\tau_{j}}{2\gamma_{2j}}\vartheta_{j}^{2}$$

(33) $$-\frac{\lambda_{j}}{\delta_{2j}}{\tilde{\varepsilon }}_{j}{\hat{\varepsilon }}_{j}=-\frac{\lambda_{j}}{\delta_{2j}}{\tilde{\varepsilon }}_{j}^{2}-\frac{\lambda_{j}}{\delta_{2j}}{\tilde{\varepsilon }}_{j}\varepsilon_{j}\leq -\frac{\lambda_{j}}{2\delta_{2j}}{\tilde{\varepsilon }}_{j}^{2}+\frac{\lambda_{j}}{2\delta_{2j}}\varepsilon_{j}^{2}$$

(34) $$-\frac{\eta_{j}}{\chi_{2j}}{\tilde{W}}_{j}^{T}{\hat{W}}_{j}=-\frac{\eta_{j}}{\chi_{2j}}{\tilde{W}}_{j}^{T}{\tilde{W}}_{j}-\frac{\eta_{j}}{\chi_{2j}}{\tilde{W}}_{j}^{T}{\hat{W}}_{j}\leq -\frac{\eta_{j}}{2\chi_{2j}}{\tilde{W}}_{j}^{T}{\tilde{W}}_{j}+\frac{\eta_{j}}{2\chi_{2j}}{\tilde{W}}_{j}^{T}{\hat{W}}_{j}.$$

With inequalities (28)–(30), it can be obtained as:

(35) $$\begin{array}{c} {\dot{V}}_{3}\leq -\frac{r\mu_{1}^{2}}{h_{a}^{2}-\mu_{1}^{2}}-\bar{k}\mu_{2}^{T}\mu_{2}-\frac{\zeta }{\sigma }\rho^{2}+\sum_{j=1}^{3}\frac{\tau_{j}}{\gamma_{2j}}\vartheta_{j}^{2}+\sum_{j=1}^{3}\frac{\lambda_{j}}{\delta_{2j}}\varepsilon_{j}^{2}+\sum_{j=1}^{3}\frac{\eta_{j}}{\chi_{2j}}{\tilde{W}}_{j}^{T}{\tilde{W}}_{j}+\sum_{j=1}^{3}\frac{\eta_{j}}{\chi_{2j}}{\tilde{W}}_{j}^{T}{\hat{W}}_{j}\;\\ +\sum_{j=1}^{3}\frac{1}{2\tau_{2j}}{\tilde{D}}_{j}^{2}+\sum_{j=1}^{3}\frac{1}{2\tau_{2j}}{\bar{\hat{D}}}_{j}^{2} \end{array}$$

Define

(36) $$\begin{array}{c} \omega =\min\{r_{1},r_{2},r_{3},{\bar{k}}_{1},{\bar{k}}_{2},{\bar{k}}_{3},\zeta \}\;\\ \sigma =\sum_{j=1}^{3}\frac{\tau_{j}}{\gamma_{2j}}\vartheta_{j}^{2}+\sum_{j=1}^{3}\frac{\lambda_{j}}{\delta_{2j}}\varepsilon_{j}^{2}+\sum_{j=1}^{3}\frac{\eta_{j}}{\chi_{2j}}{\tilde{W}}_{j}^{T}{\tilde{W}}_{j}+\sum_{j=1}^{3}\frac{1}{2\tau_{2j}}{\tilde{D}}_{j}^{2}+\sum_{j=1}^{3}\frac{1}{2\tau_{2j}}{\tilde{\hat{D}}}_{j}^{2} \end{array}$$

Then (31) is equivalent to:

(37) $${\dot{V}}_{3}\leq -\omega V_{3}+\sigma$$

Multiply $e^{\omega t}$ on the both sides of the Equation (32) and integrate in the interval [0,t] to get:

(38) $$0\leq V_{3}\left(t\right)\leq \left[V_{3}\left(0\right)-\frac{\sigma }{\omega }\right]e^{-\omega t}+\frac{\sigma }{\omega }$$

From (33), it can be concluded that $\bar{V}\left(t\right)$ is bounded. Therefore, the tracking error $\mu_{1}$, signals $\mu_{2}$, observer error $\tilde{D}$, adaptive parameters ρ, parameter estimates $\hat{\theta }$, $\hat{\varepsilon }$ and the estimated RBFNN weight vector, ${\tilde{W}}_{j}$ can converge to a compact set, $\Omega =\left[V_{3}\left(0\right)-\frac{\sigma }{\varpi }\right]e^{-\omega t}+\frac{\sigma }{\varpi }$ can converge to a compact set.

**Remark 2.**  *To obtain higher approximation accuracy, it is typical to utilize a large number of neurons in RBFNN control design. Nevertheless, the approximation accuracy *(25)* can be fine-tuned by the parameter *(23)*, which removes the requirement for a large number of neurons in RBFNNs.*

## 3. Simulation Verification of RBFNN-Based Adaptive Robust Control

Consider the desired tracking trajectory defined by the following continuous functions along the XYZ-axis, where all three functions are smooth and differentiable.

$$y_{d}={\left[y_{d1},y_{d2},y_{d3}\right]}^{T}={\left[-0.5\cos\left(\pi t\right),0.3-0.05\cos\left(\pi t\right),0.35\sin\left(\pi t\right)\cos\left(0.5t\right)\right]}^{T}$$

Three kinds of external disturbance are considered in the simulation.

The initial values in the FOG are given as Table 1.

Simulation 1: When the external disturbance is $D={\left[D_{1},D_{2},D_{3}\right]}^{T}=[2\cos\left(3t\right),$${2\sin\left(5t\right),2\sin\left(5t\right)]}^{T}$, where each function are is smooth and differentiable.

**Figure 3:**
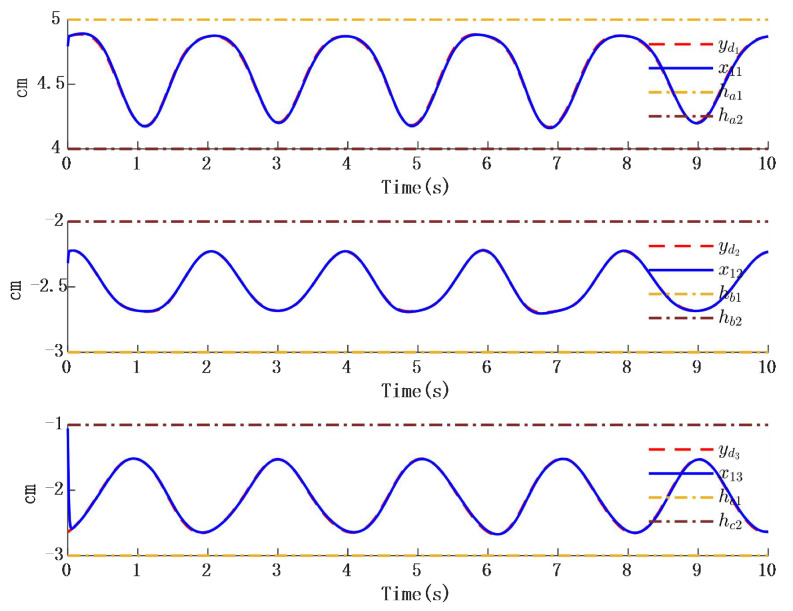
Time response of position tracking error.

**Figure 4:**
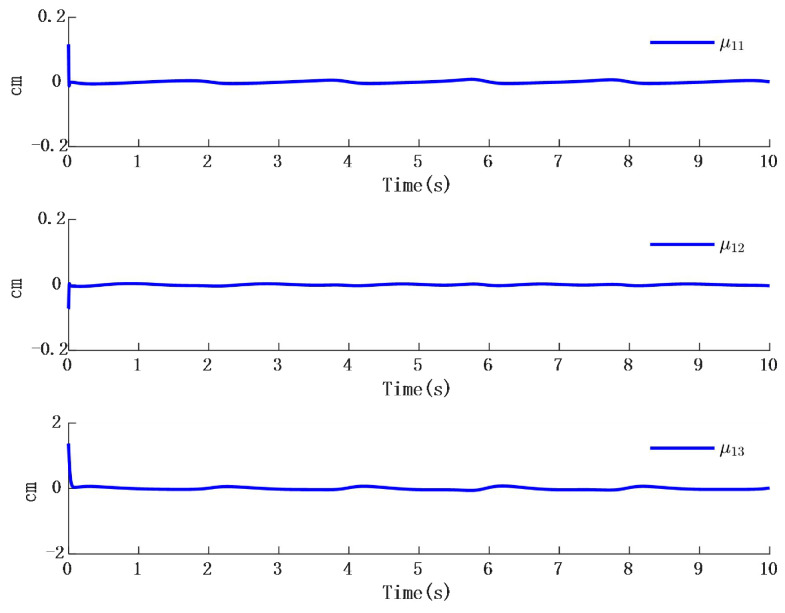
Time response of position tracking error.

**Figure 5:**
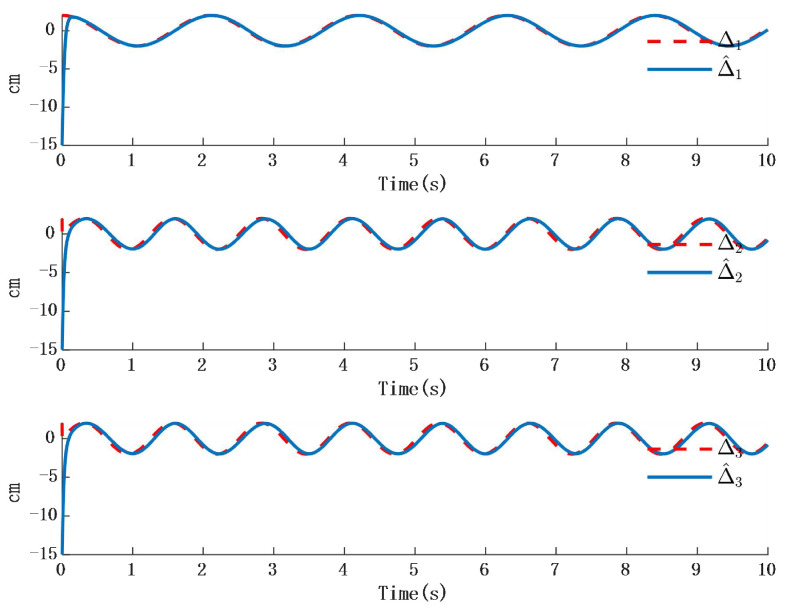
Time response of actual disturbance and disturbance observer error.

**Figure 6:**
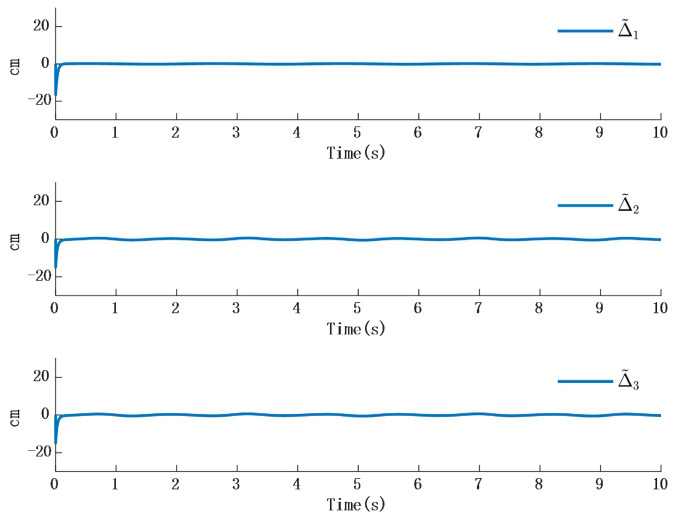
Time response of actual disturbance and disturbance observer error.

**Figure 7:**
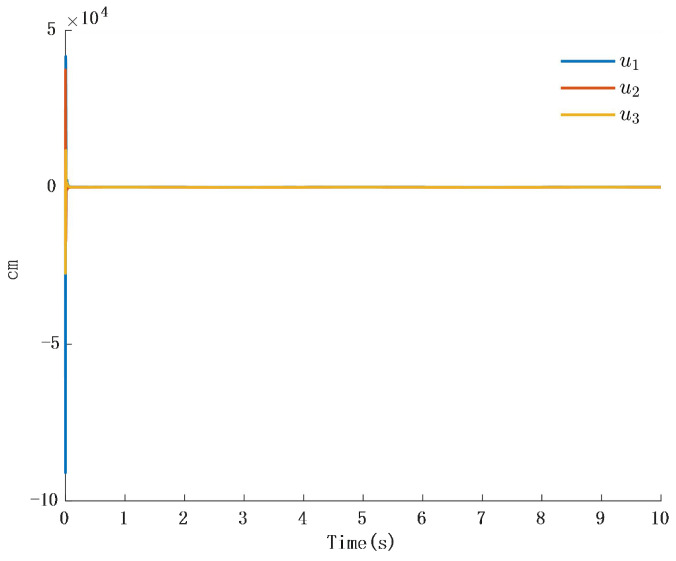
Time response of actual disturbance and disturbance observer error.

**Figure 8:**
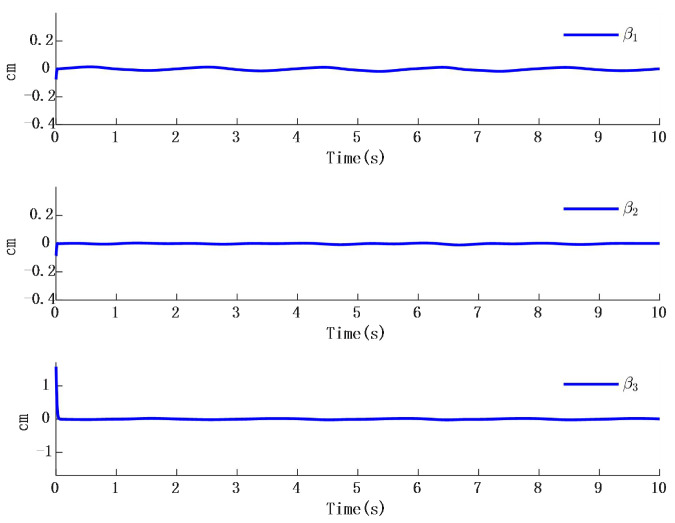
Time response of the controller β.

**Figure 9:**
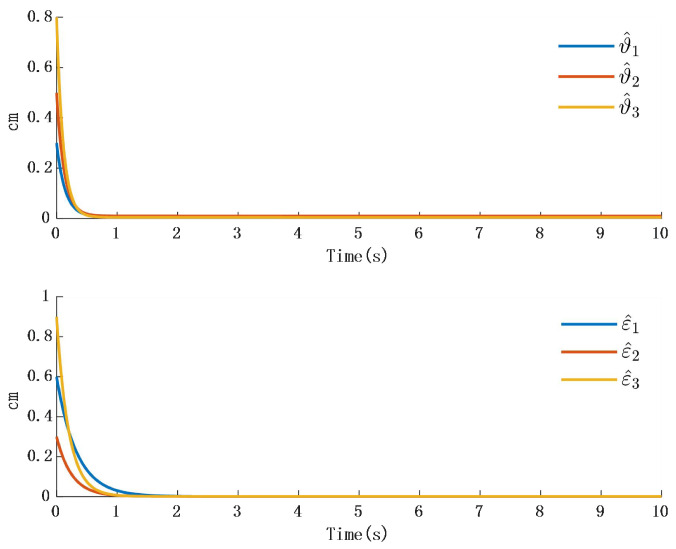
Time response of adaptive parameters.

In Figure 2, the output of the FOG control system (1) effectively tracks the desired reference signal, and the time response of the tracking error is shown in Figure 3. The simulation results indicate that the tracking error converges. The actual disturbance of the system and the simulation results of the disturbance observer are shown in Figure 4, while the time response of the observation error is presented in Figure 5. These results demonstrate that the disturbance observer designed in this study achieves high-precision observation. The corresponding controller and the parameter adaptation laws are shown in Figure 6, Figure 7, Figure 8 and Figure 9. Figure 6 illustrates that the overall time-varying trend of the controller remains stable. Figure 7 shows the time response of the virtual controller. Figure 8 depicts the time response of the adaptive parameters within the controller. The variation of the Lipschitz adaptive parameters in Figure 8 indicates the existence of a series of suitable small constants. In Figure 9, the nonzero parameters within the controller can automatically adjust online and reach appropriate values, thereby satisfying the optimal approximation performance of the RBFNN. The approximation accuracy of the RBFNN can automatically adjust and asymptotically approach zero. These simulation results demonstrate that the RBFNN adaptive controller designed in this study effectively achieves the desired system tracking and high-precision tracking and observation of unknown external disturbances.

**Figure 10:**
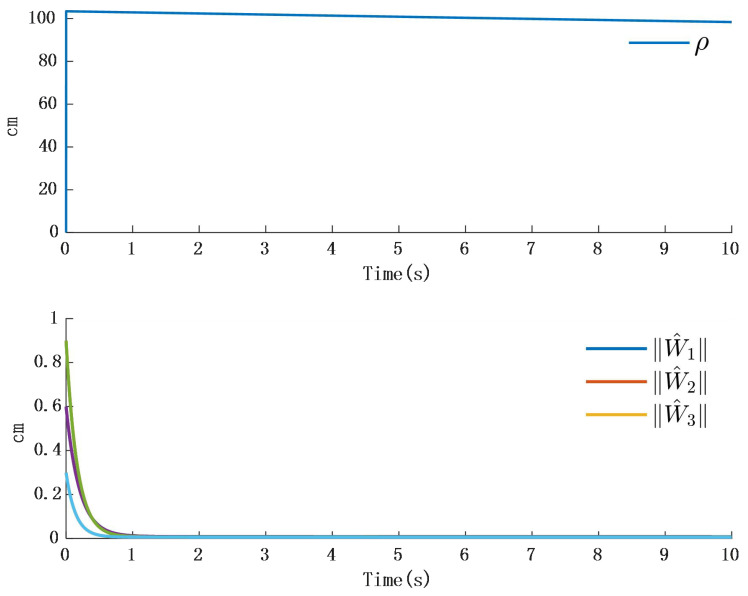
Time response of adaptation in the controller.

**Figure 11:**
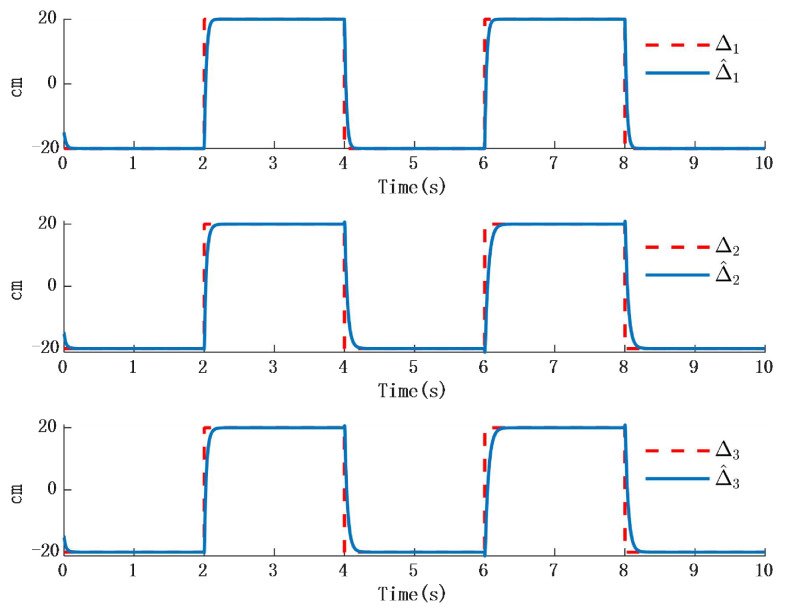
Step time response and time response of the observer.

Simulation 2: When the external disturbance is $D={\left[D_{1},D_{2},D_{3}\right]}^{T}$ a step response as shown in Table 2, where the three external disturbances along the XYZ-axis are modeled as non-differentiable piecewise functions, commonly named as square wave interference, the simulation results are presented in Figure 10 and Figure 11:

The simulation results in Figure 10 illustrate the comparison between the designed disturbance observer and the actual disturbance under step responses occurring at different time intervals. It can be known from Figure 9 that the time response of the observer designed in this paper is almost the same as that in the actual interference situation. Since the time response in the actual situation is also affected by other factors, such as environmental temperature, etc., a certain delay will occur in the system when converting between high and low levels. However, in the stable state, the observer designed in this paper is consistent with the time response characteristics of the actual situation. Therefore, it will not have an impact on the performance of the system. Figure 11 presents the time response of the error between the actual step disturbance and the observed step disturbance. It is obvious that the error between the actual step response and the observed step response time is consistent. It can therefore be concluded that the designed disturbance observer achieves effective observation of external disturbances even when the disturbances take the form of step signals.

Simulation 3: When the external disturbance is $D={\left[D_{1},D_{2},D_{3}\right]}^{T}$. In this case, each function includes a ramp signal as described below, where the magnitude of the ramp signal increases linearly over time.

$$D_{j}=\left\{\begin{array}{cc} 3t, & 0\leq t<2\\ 3t-2, & 2\leq t<4\\ 3t-4, & 4\leq t<6\\ 3t-6, & 6\leq t<8\\ 3t-8, & 8\leq t<10 \end{array}\right.$$

**Figure 12:**
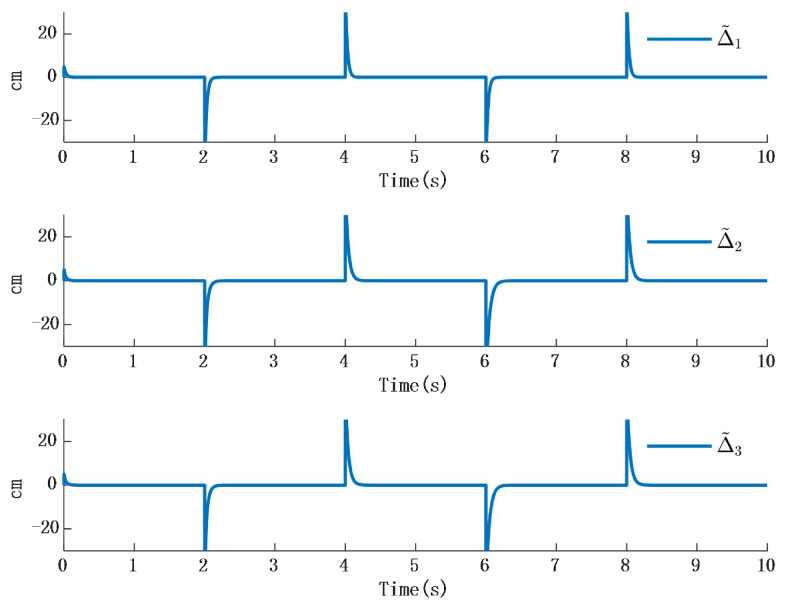
Time response of the error between step response and observer.

**Figure 13:**
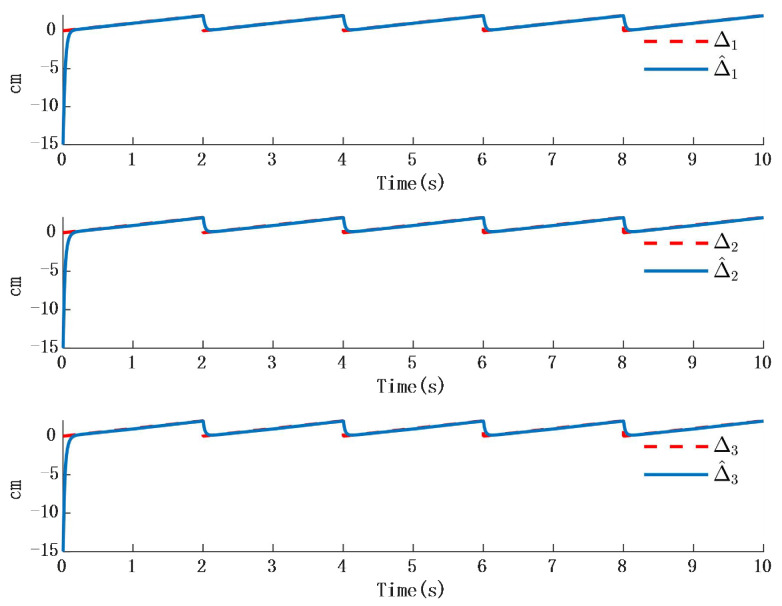
Time response of slope and observer.

**Figure 14:**
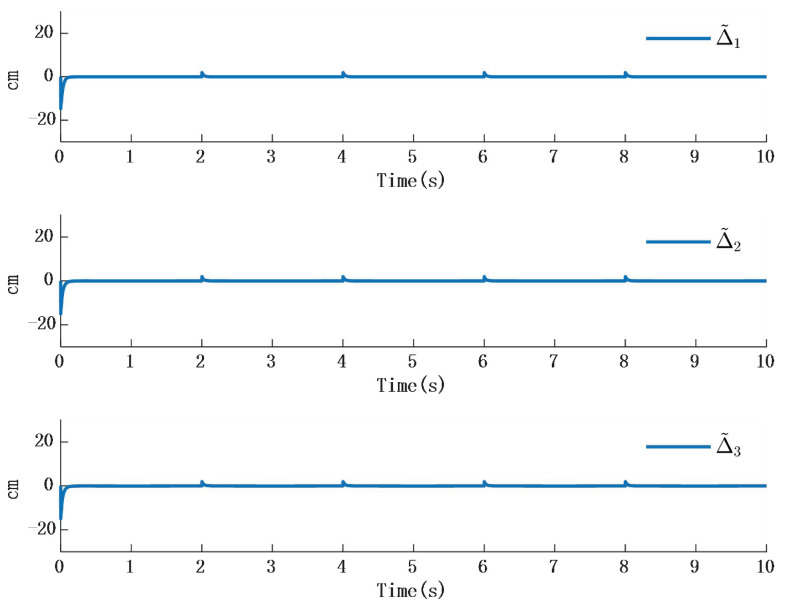
Error time response of slope and observer.

Figure 12 and Figure 13 shows the simulation results; Figure 14 shows error time response of slope and observer:

In Figure 12, the time response of the observer aligns with the external disturbance when the disturbance takes the form of a ramp signal. Figure 13 shows that the time response of the error between the ramp disturbance and the observer converges to zero.

From the simulation results of three different types of external disturbances, it can be concluded that the proposed design method enables the output of the FOG system (1) to effectively track the desired reference signal. Moreover, the external disturbances can be effectively observed through the designed observer. The unknown dynamic model within the system can be effectively approximated using the designed RBFNN, with the approximation accuracy being adaptively regulated through the controller parameters, significantly improving the approximation precision. Furthermore, all signals in the closed-loop system are ensured to achieve uniform ultimate boundedness.

**Remark 3.**  *In the first simulation, a smooth and differentiable desired trajectory along the XYZ-axis. The results show that the system can achieve accurate tracking performance, and the disturbance observer error converges to zero. In the second simulation, a step-like external disturbance, which is non-differentiable at the inflection point due to its piecewise nature. This represents a different class of disturbance compared to the first case. Finally, in the third simulation, a piecewise increasing function as the external disturbance, which presents a more complex and challenging scenario than the previous two cases. Despite these varying levels of complexity such as increasing, the simulation results consistently demonstrate good tracking performance, and the disturbance observer effectively estimates and compensates for the unknown external disturbances.*

**Remark 4.**  *This study models the dynamical equation of the Digital closed-loop FOG, differing from other models that linearize the nonlinear terms. In this work, the RBFNNs to approximate the unknown nonlinear terms. Therefore, comparisons with other methods for same class of Digital closed-loop FOG is the main research work in future. because of the unique model of the digital closed-loop FOG.*

**Remark 5.**  *The main focus of this work is on the design of RBFNN adaptive control theory for FOG. Experimental testing is a key task due to more complex factors such as hardware and others, it is our main work in future research.*

## 4. Conclusions

A new RBFNN-based adaptive robust control technique is proposed for high-precision angular position and velocity tracking of FOG under large dynamic unknown external disturbances. The designed controller not only relaxes the limitations imposed by the finite domain of the RBFNN but also enables online adjustment based on the desired control accuracy. Compared to the simulation results under the different unknown external disturbance, the RBFNN-based adaptive robust control technique demonstrates the ability to achieve high-precision tracking and effective disturbance observation in the presence of unknown external disturbances. Indeed, there are numerous complex factors that can influence the system, such as hardware implementation, unbounded disturbances, and integration with multi-sensor systems. These various elements pose challenges in practical applications. The research conducted can provide significant importance for enhancing the performance of high-dynamic FOG-based strapdown inertial navigation systems.

## Figures and Tables

**Table 1:** Initial values in the fiber optic gyro control system.

Parameter	Value	Parameter	Value	Parameter	Value
$\phi_{F1}$	−0.4	$\phi_{F2}$	0.22	$\phi_{F3}$	−0.15
$\phi_{s1}$	−π	$\phi_{s2}$	π	$\phi_{s3}$	−0.5π
$k_{11}$	3	$k_{12}$	3	$k_{13}$	3
$k_{21}$	10	$k_{22}$	10	$k_{23}$	10
α	100	*L*	10	$h_{a1}$	5
$h_{b1}$	−2	$h_{b2}$	−3	$h_{c1}$	−1
$\tau_{1}$	100	$\tau_{2}$	200	$\tau_{3}$	300
$\rho_{1}\left(0\right)$	0.6	$\rho_{2}\left(0\right)$	0.9	$\rho_{3}\left(0\right)$	0.3
$\gamma_{21}$	0.001	$\gamma_{22}$	0.002	$\gamma_{21}$	0.003
$r_{1}$	3	$r_{2}$	3	$r_{3}$	3
$\delta_{11}$	0.002	$\delta_{22}$	0.004	$\delta_{23}$	0.006
${\bar{k}}_{1}$	100	${\bar{k}}_{2}$	200	${\bar{k}}_{3}$	300
$\lambda_{1}$	250	$\lambda_{2}$	350	$\lambda_{3}$	450
ξ	20	σ	10	ρ(0)	0.1
$\vartheta_{1}\left(0\right)$	0.6	$\vartheta_{2}\left(0\right)$	0.9	$\vartheta_{3}\left(0\right)$	0.3
$\varepsilon_{1}\left(0\right)$	0.5	$\varepsilon_{2}\left(0\right)$	0.7	$\varepsilon_{3}\left(0\right)$	0.4

**Table 2:** Disturbance values produced in time 0 to 10.

Time (Second)	0≤t≤2	2<t≤4	4<t≤6	6<t≤8	8<t≤10
$D_{1}$	−20	20	−20	20	−20
$D_{2}$	−20	20	−20	20	−20
$D_{3}$	−20	20	−20	20	−20

## Data Availability

The original contributions presented in this study are included in the article. Further inquiries can be directed to the corresponding author.
